# Towards the Integrated Management of *Fusarium* Wilt of Banana

**DOI:** 10.3390/jof10100683

**Published:** 2024-09-29

**Authors:** Guy Blomme, George Mahuku, Elizabeth Kearsley, Miguel Dita

**Affiliations:** 1 The Alliance of Bioversity International and CIAT, c/o ILRI, Addis Ababa P.O. Box 5689, Ethiopia; 2 The International Institute of Tropical Agriculture (IITA), Kampala P.O. Box 7878, Uganda; 3 BlueGreen Labs, 9120 Melsele, Belgium; kearsleyelizabeth@gmail.com; 4 The Alliance of Bioversity International and CIAT, Sao Paulo 71600-500, Brazil; miguel.dita@gmail.com

## 1. The Topic

This Special Issue contains a selection of papers dealing with *Fusarium* wilt of banana (FWB), with a special focus on the *Fusarium* strain Tropical Race 4 (TR4), and explores (1) options for effective integrated management strategies, (2) the detection and development of disease-resistant cultivars, and (3) the distribution and diversity of the pathogen. 

*Fusarium* wilt of banana caused by *Fusarium oxysporum* f. sp. *cubense* (Foc) Tropical Race 4 (TR4) is considered to be one of the greatest threats to the banana sector worldwide [1,2]. TR4 was first identified in Taiwan in 1989 [3] and over subsequent decades spread to countries within the greater Mekong subregion and to Australia [4,5]. In 2013, the first official report outside of Southeast Asia was made in the Middle East [6]. TR4 has since continued to spread, with new incursions reported on the Indian subcontinent, southern Africa, and even Europe [7]. The outbreak in Colombia [8] was the first observation of TR4 in Latin America, taking the disease to the pandemic level. New incursions continue to be recorded, with the most recent reports in Turkey [9], Mayotte [10], Peru [11], and Venezuela [12].

The TR4 strain is extremely virulent toward many banana cultivars, including the economically important Cavendish cultivars. The Cavendish subgroup, mostly grown in large-scale monoculture plantations, amounts to almost half of the global banana production and the bulk of global export and trade [2], which could be devastated by further spread of TR4. Many other banana varieties cultivated in local and often more diverse production systems, important for local food security and income, are also TR4-susceptible and would be severely impacted.

This Special Issue brings together the state-of-the-art and knowledge on different aspects of integrated management of FWB. Together, the contributed studies provide a comprehensive overview of current disease management options and move the global efforts of tackling this devastating banana disease forward.

## 2. Background

The use of banana germplasm resistant to TR4 is the only real strategy to successfully manage this disease [13]. This strategy mirrors the crisis of Foc Race 1 (R1) impacting the susceptible cultivar Gros Michel, when the global banana production systems successfully shifted to the R1-resistant Cavendish subgroup. Currently, there are no suitable alternative dessert banana varieties resistant to Foc TR4 that could replace Cavendish. 

Banana breeding is a slow process that can take decades before presenting desirable results. Tolerance and resistance to TR4 have been identified in several bred hybrids, specifically several FHIA clones, but these unfortunately lack desirable properties for producers and consumers alike, such as taste or cultivation and post-harvest issues [14,15]. Local production systems could potentially rely on some TR4-resistant hybrids if local demand allows for it, but upscaling to the global market is currently not possible.

Genetic modification of bananas, through the use of trans- and cis-genic approaches, has resulted in resistance to Foc in commercial banana cultivars, including in some Cavendish cultivars, without losing other desired properties [16,17]. Unfortunately, genetically modified products still face a lot of scrutiny from regulators and consumers, with concerns from health and environmental perspectives, despite their proven safety [18].

Various TR4-tolerant Cavendish somaclones, selected from tissue culture-derived plantlets, have emerged over the past decade, with ‘Formosana’ [GCTCV-218] now replacing TR4-affected Cavendish fields in, e.g., Taiwan, the Philippines, and Mozambique [19]. This somaclone performs well when soil inoculum levels are low at the time of field planting.

The management of FWB TR4 is therefore still highly reliant on prevention, excluding the entry of the pathogen into disease-free areas, and containment, halting the spread from infected areas. Foc, however, spreads easily, mostly through the movement of planting material, contaminated soil, and water, and effective exclusion and quarantine are difficult, especially when dealing with large numbers of small-scale farms.

Prevention relies on comprehensive border, pre-border, and on-farm preventive measures. To ensure this, strict biosecurity protocols are essential, optimally within a regulatory framework of national biosecurity legislation and international agreements. Once Foc TR4 is introduced, containment measures need to be put in place, and the distribution of the pathogen and its most likely spread need to be determined.

However, Foc TR4 cannot be eradicated from soils once the pathogen has been introduced. The soilborne pathogen has been shown to persist in soils for decades, even in the absence of a suitable host. In addition to the establishment of a tolerant somaclone, or in the absence of TR4-tolerant/resistant banana cultivars, TR4 management should focus on integrating available disease management strategies [1] that reduce the buildup of Foc TR4 inoculum in the soil and minimize the further spread of the pathogen. Management strategies vary depending on regional conditions but are generally focused on optimizing soil health and suppressing the pathogen. Such strategies include the application of organic amendments and biocontrol agents, the use of inorganic fertilizers, and introducing specific agronomic practices such as crop rotation and the use of cover crops [1].

The papers of this Special Issue cover a range of topics inherent to building effective integrated disease management of *Fusarium* wilt, including identification and screening for resistance [20,21], management of the disease through soil treatments (Segaru et al., 2021) and organic amendments [22,23,24], and the impact of infected plant removal using herbicide on the Foc inoculum [25]. In addition, the particularities of pathogen spread as affected by weevils [26], via water [27], and garden tool vectored soil spread [28] are presented. 

The genetic diversity in Indian-origin *Fusarium* strains is investigated by Thangavelu et al. (2021) [29] and Thangavelu et al. (2022) [30], while the current geographic distribution of FWB in Laos and Vietnam [31] is also presented.

## 3. The Research Papers

### 3.1. Integrated Management Strategies

In-depth knowledge of Foc TR4-resistant or highly tolerant *Musa* cultivars is needed to devise cultivar replacement strategies in highly affected regions. Through a systematic literature review, Rocha et al. [20] identified the improvements made on the resistance of *Musa* spp. to FWB in the last decade. They identified the sources of resistance related to Foc R1 and TR4 in the current literature, and discussed the main methods and protocols used to obtain and characterize resistance to FWB in detail. The authors provided comprehensive lists of germplasm with resistance traits, report genes associated with resistance, genes used for transgenesis, and more. Mintoff et al. [21] built on the body of literature reviewed by Rocha et al. [20], screening 24 *Musa* accessions for their resistance to TR4. They identified nine highly resistant cultivars and an additional four showing minor symptoms, among these cultivars of the Cavendish subgroup, FHIA hybrids, and Pisang Gajih Merah. Newly identified cultivars resistant to TR4 bring us a step closer to finding acceptable solutions for banana producers and consumers worldwide.

In the meantime, targeted disease management strategies can alleviate the impact of *Fusarium* wilt. Managing soil properties to reduce the predisposition of *Musa* to the disease or practices that reduce the inoculum load in the soil can present options for continued banana cultivation in affected areas. Experimental work by Segaru et al. confirmed the role of soil management on FWB under field conditions. A higher soil pH was linked to reduced plant mortality with improved plant development. Plant mortality did, however, increase after several crop cycles, and the authors highlighted that additional disease management strategies are imperative. 

The use of beneficial and antagonistic microorganisms for biological control shows good potential as an effective approach to control FWB [32]. The *Bacillus* group, amongst a few others, has been identified as a potentially effective biocontrol agent against TR4. Li et al. [22] and Fan et al. [23] investigated and identified, respectively, five and two *Bacillus* strains strongly inhibiting TR4. *Bacillus* biocontrol marker genes were identified in both studies and evaluated to assess their biocontrol potential. Li et al. [22] specifically developed a reverse transcription quantitative PCR assay to characterize biocontrol gene expression profiles and patterns among *Bacillus* strains, suggesting different mechanisms are involved in biocontrol of TR4. Similarly, Fan et al. [23] highlighted the diversity in *Bacillus* biocontrol mechanisms by detecting the *sboA* marker gene exclusively in a specific strain.

The potential of the spent substrate of the mushroom *Pleurotus ostreatus* to suppress Foc was investigated by Ocimati et al. [24] Using both in vitro trials and greenhouse potted plant experiments, the authors provided evidence of the spent substrate suppressing Foc R1, with potential competition, antibiosis, and predation mechanisms involved. 

In affected areas, the removal and eradication of TR4-infected plants form an integral part of disease control. The method of plant eradication can influence pathogen survival and sporulation. As such, Anderson and Aitken [25] examined the production of Foc STR4 inoculum following the treatment of infected plants with herbicides and fungicides. The authors demonstrated that treatments with fungicides did not prevent Foc sporulation, while specific herbicides even hastened the colonization by Foc in senescent banana plant tissue with hyphae and the production of micro- and macroconidia. This study highlights the important finding that plants treated with fungicide and/or herbicide remain an important or even increased source of pathogen inoculum in the field.

Halting, or at least slowing down, the spread of the TR4 pathogen depends on strict containment protocols and biosecurity measures. Pathogen containment by disinfecting tools, vehicles, shoes, and any vector that can carry contaminated soil particles with proper disinfectants is an imperative part of these protocols. The use and efficacy of commercial quaternary ammonium compound (QAC) products against Foc TR4 was tested by Izquierdo-García et al. [28]. They identified that the presence of soil can reduce the efficacy of some disinfectants, but highlight which disinfectants show reliable efficacy against Foc microconidia, macroconidia, and chlamydospores under varying local conditions, including disinfectant contact times and concentrations and the presence of organic matter. The authors highly recommend the removal of soil before disinfection. Their results can improve biosecurity measures by optimizing the disinfection of vehicles, equipment, boots, and personal hygiene facilities, among others.

Natural dispersal agents or processes, such as erosion, wind, runoff, and animals, are more difficult to detect and control. A good understanding of TR4 dispersal mechanisms can improve both exclusion and containment strategies. Water movement, however, is a particularly concerning dispersal mechanism, as infested water can rapidly contaminate disease-free areas. Ullah et al. [27] demonstrated that the pathogen can survive for at least 120 days in agitated water, both in the absence and presence of soil, but reduced survival times were recorded in stagnant water. The authors also confirmed that most Foc spores settle at the bottom of the water body, although they observed much longer settling times, up to 30 days, than was previously recorded. The authors highlight the need to treat water before using it for irrigation, even when collected at the surface. They recommend the use of peracitic acid products after the filtration of organic matter for effective water treatment.

At field level, the involvement of two insect pests of banana, the weevil borer and the false weevil borer, on the dispersal of Foc was investigated by Heck et al. [26] under field conditions. The authors observed a higher FWB incidence in fields with a higher weevil borer population and suggest that these weevil borers potentially act as a dispersal agent or predispose the banana plants to infection by Foc. Management of weevil borer populations in banana fields can help reduce local dispersal of Foc.

### 3.2. Diversity in Fusarium oxysporum f. sp. cubense 

The differentiation of Foc strains is an important aspect for detecting and mitigating new outbreaks. The study of Thangavelu et al. [29] on the genome assembly of Indian-origin Foc R1, STR4, and TR4 provides some interesting findings. Protein-coding genes of Foc genomes associated with biological processes, cellular components, and molecular functions were annotated. However, the authors also recorded a large number of protein-coding genes of STR4 and TR4 with unknown functions or features, potentially related to genes undergoing rapid evolution, and highlighted their significance in predicting the virulence and pathogenicity of these Foc strains. The authors further investigated mechanisms of the major pathogenicity-related protein families involved in increasing the pathogenicity and virulence of organisms and assessed variations in the organization of genome assembly and virulence-associated genes.

A high diversity In genomic organization and gene profiles of Indian Foc strains was also observed by Thangavelu et al. [30] The authors focused on developing race-specific markers that allow for target amplification in the characterized highly virulent Foc isolates and do not show any cross-amplification to other Foc races or VCGs, other *Fusarium* species, or non-pathogenic *Fusarium oxysporum* isolates. The developed markers are intended for the accurate diagnosis and detection of the Indian Foc races, which can aid in the development of effective disease management.

Characterizing the geographical distribution of *Fusarium* strain diversity can clarify disease dynamics in time and space and inform on necessary management strategies. As such, investigating the geographic distribution of Foc strains can help identify how the pathogen has spread and help identify ways to halt this spread. Across Laos and Vietnam, Chittarath et al. [31] observed a distinct geographical spread of Foc TR4 and R1 vegetative compatibility groups and the type of banana production systems affected. For example, in Laos, large-scale Cavendish plantations owned by or with Chinese company involvement regularly imported planting material from China, and these materials were often infected with TR4, while in Vietnam, TR4 was mostly observed in small-holder farms and backyard gardens. This calls for geographically distinct management strategies.

### 3.3. Way Forward

Currently, there is no silver bullet to manage FWB TR4. Limiting the spread of TR4 requires effective mobilization and collaboration among global, regional, national, and local stakeholders to strengthen plant quarantine, surveillance, and containment efforts. Three dimensions: (a) halting new disease outbreaks, (b) preventing pathogen spread in affected areas, and (c) finding long-term solutions, such as resistant cultivars for sustainable disease management, are often considered according to the prevalent scenario. The use of beneficial microorganisms has received increasing attention for the management of FWB. However, holistic insights into the soil and plant microbiome and its role in creating disease-suppressive soils need to be obtained. Translating the immense amount of information already generated into practical and site-specific bio-based approaches is overarching. To achieve this, targeted research questions need to be formulated and further validated in collaboration with field-based stakeholders. Finally, effective management of FWB will only be possible through the incorporation of genetic resistance into integrated disease management approaches. Developing banana genotypes that are highly resistant to FWB and commercially acceptable is, however, not an easy task. Alternative technologies to conventional breeding, such as CRISPR and mutation breeding, may speed up the process and are hoped to deliver solutions over the coming years. Governments of TR4-affected countries should also develop legal frameworks to facilitate the import and assessment of promising genotypes. These efforts should be backed with more investments from the private sector, including not only growers but all stakeholders along the value chain.
